# Abnormal Nerve Conduction Study Findings Indicating the Existence of Peripheral Neuropathy in Multiple Sclerosis and Neuromyelitis Optica

**DOI:** 10.1155/2013/847670

**Published:** 2013-11-07

**Authors:** Yoko Warabi, Mikihiro Yamazaki, Toshio Shimizu, Masahiro Nagao

**Affiliations:** Department of Neurology, Tokyo Metropolitan Neurological Hospital, 2-6-1 Musashidai Fuchu, Tokyo 183-0042, Japan

## Abstract

*Objective*. Chronic inflammatory demyelinating polyneuropathy (CIDP) has been reported in patients with multiple sclerosis (MS). However, there have been limited reports of peripheral neuropathy as a complication of neuromyelitis optica (NMO). In this paper, we showed the characteristics and differences between peripheral neuropathy as a complication of MS and NMO. *Method*. We analyzed a series of 58 MS and 28 NMO patients and evaluated nerve conduction studies (NCS) in 21 MS and 5 NMO patients. *Results*. Six of the 58 MS and 3 of the 28 NMO patients revealed abnormal NCS findings. Three (5.2%) of the 58 MS patients fulfilled the criteria for CIDP. One (3.6%) of the 28 NMO patients showed peripheral neuropathy at the same time of NMO relapse, although CIDP was not seen in NMO. The other 5 (3 MS and 2 NMO) patients were complicated with neuropathy caused by concomitant diabetes mellitus and Sjögren's syndrome. *Conclusion*. Frequency of abnormal NCS findings might exhibit no significant difference between MS and NMO, although the cause and pathophysiology of peripheral neuropathy were different in MS and in NMO. There might be a group of NMO who were affected simultaneously in the central and peripheral nervous tissues.

## 1. Introduction

Peripheral neuropathy and chronic inflammatory demyelinating polyneuropathy (CIDP) have been reported in patients with multiple sclerosis (MS) [1, 2], and common antigens between the central nervous system (CNS) and peripheral nervous system (PNS), such as myelin basic protein (MBP) and myelin-associated glycoprotein (MAG), were suspected to be pathogens of the coexisting MS and CIDP [3]. Neuromyelitis optica (NMO) is another inflammatory demyelinating disease of the CNS which is characterized by lesions confined to the optic nerve and spinal cord, especially longitudinally extensive spinal cord lesions [4], antiaquaporin-4 (AQP-4) autoantibody seropositivity [5], and astrocytic impairment associated with the loss of AQP-4 in NMO lesions [6]. There have been limited reports about the characteristics of peripheral neuropathy as a complication of NMO [7, 8]. In this paper, we evaluated the electrophysiological changes with nerve conduction studies (NCS) in MS and NMO patients and showed the characteristics and differences between peripheral neuropathy as a complication of MS and NMO.

## 2. Patients and Methods

We retrospectively analyzed the medical records including NCS findings and magnetic resonance imaging (MRI) findings of a series of Japanese MS and NMO patients admitted to our hospital between 2010 and 2011. Fifty-eight (67%) MS patients and 28 (33%) NMO patients had been admitted in this period. This ratio of MS and NMO patients is consistent with the Japanese patients, because there is a consensus that NMO comprises about one third of the Japanese CNS inflammatory demyelinating diseases [9]. Then, we identified 21 MS patients and 5 NMO patients who were suspected of having peripheral neuropathy because they showed neurological findings such as a reduced deep tendon reflex or sensory disturbance of the peripheral extremities, and they were evaluated by NCS. For each nerve, the electrophysiological data are considered to be abnormal if they are not within 2.0 standard deviations (SD) from mean for healthy age-matched controls in our hospital. We used the revised McDonald criteria for MS [10] and revised Wingerchuk criteria for NMO and NMO spectrum disorders [11, 12], and the European Federation of Neurological Societies/Peripheral Nerve Society (EFNS/PNS) electrodiagnostic criteria for CIDP [13] for the diagnosis of MS, NMO, and CIDP, respectively. 

## 3. Results

Six (10.3%) of the 58 MS and 3 (10.7%) of the 28 NMO patients revealed abnormal NCS findings.  Table 1 shows the clinical characteristics associated with the CNS demyelinating diseases of the 9 (6 MS and 3 NMO) patients. All of the 3 NMO patients showed anti-AQP-4 autoantibody seropositivity. As disease-modifying therapy for preventing relapses, one MS patient (Patient 3) was treated with interferon beta-1b, one NMO patient (Patient 7) was treated with azathioprine (100 mg/day), and one NMO patient (Patient 9) was treated with oral prednisolone (7.5 mg/day). For the treatment of MS and NMO relapses, all of the 9 patients received intravenous methylprednisolone (IVMP), and one NMO patient (Patient 7) was treated with additional intravenous immune globulin (IVIg).

Table 2 shows the characteristics associated with the peripheral neuropathy of the 9 patients. Three (5.2%) of the 58 MS patients were complicated with CIDP. Two MS patients (Patient 1 and 2) fulfilled the EFNS/PNS electrodiagnostic criteria for definite CIDP, and one MS patient (Patient 3) fulfilled the criteria for probable CIDP. All three CIDP patients complicated with MS (Patients 1, 2, and 3) showed conduction block and nerve conduction velocity slowing of the compound muscle action potential (CMAP) and the sensory nerve action potential (SNAP).  Moreover, one patient with probable CIDP complicated with MS (Patient 3) showed temporal CMAP dispersion. Although the remaining 6 MS and NMO patients showed NCS abnormality, indicating the existence of peripheral neuropathy, that is, nerve conduction velocity slowing, conduction block, prolonged duration, low amplitude, and reduced F-wave occurrence, they did not fulfill the EFNS/PNS electrodiagnostic criteria for definite or probable CIDP. The rest 3 MS patients who did not have CIDP (Patients 4, 5, and 6) were complicated with type 2 diabetes mellitus (DM). Their results of NCS were characterized by low amplitudes in lower extremities which were suspected to be the characteristics of DM neuropathy coexistence. 

Three NMO patients were complicated with peripheral neuropathy. However, typical CIDP was not seen in NMO. Peripheral neuropathy as a complication of NMO showed two types of clinical course. One (3.6%) of the 28 NMO patients showed peripheral neuropathy at the same time of NMO relapse. Patient 7 showed normal NCS results at the time of his first NMO attack. He experienced a relapse of NMO in the medulla oblongata and the first segment of cervical cord at one year after the onset of NMO, and he entered tetraplegic state. At that time, he was suspected of having peripheral neuropathy because he showed a reduced deep tendon reflex. He showed prolonged CMAP duration in tibial nerves and low CMAP amplitude in median nerves determined by NCS. He was treated with IVMP and IVIg. Two years later, his NMO recovered, he was able to walk. Conversely, another 2 NMO patients (Patients 8 and 9) gradually developed walking disturbance without remarkable relapses of NMO at the time of more than 10 years after the onset of NMO. These two NMO patients were complicated with Sjögren's syndrome. 

All of the peripheral neuropathies occurred more than one year after the onset of MS and NMO, and five (56%) of the 9 peripheral neuropathies occurred more than 10 years after the onset of MS and NMO. In the evaluation of CNS demyelinating lesions in MRI of the 9 patients with NCS abnormality, all 9 (100%) showed spinal cord lesions, 6 (67%) showed brainstem lesions, 5 (56%) showed cerebral lesions, and 2 (22%) showed optic nerve lesions (Table 1). 

## 4. Discussion

In this analysis, 6 (10.3%) of the 58 MS and 3 (10.7%) of the 28 NMO patients revealed abnormal NCS findings indicating the existence of peripheral neuropathy. Three (5.2%) of the 58 MS patients fulfilled the criteria for CIDP. One (3.6%) of the 28 NMO patients showed peripheral neuropathy at the same time of NMO relapse, although CIDP was not seen in NMO. The other 5 (3 MS and 2 NMO) (5.8%) of total of the 86 MS and NMO patients were complicated with type 2 DM and Sjögren's syndrome. Their results of NCS and clinical course were suspected to have the characteristics of neuropathy caused by these concomitant conditions. These results show that the frequency of abnormal NCS findings indicating the existence of peripheral neuropathy might exhibit no significant differences between MS and NMO, although the cause and pathophysiology of peripheral neuropathy were different in MS and in NMO. 

Our MS patients were complicated with typical CIDP as in many previous reports [1, 2]. MS is a disease mediated by T-cell immunity [14, 15], and CIDP as a complication of MS is also believed to be mediated by T-cell immunity specific for myelin antigens [16, 17]. Peripheral and central myelin have different protein compositions, but they share some proteins such as MBP and MAG [3]. Therefore, an abnormal autoimmune response against a common antigen might cause both MS (CNS demyelination) and CIDP (PNS demyelination) [18]. However, if a common antigen is the pathogen, MS and CIDP would occur at the same time. In this study, most of the peripheral neuropathies occurred more than 10 years after the onset of MS. Thus, we assume that MS and peripheral demyelinating neuropathy were not caused by a T-cell reaction specific for a shared antigen and that peripheral demyelinating neuropathy as a complication of MS was caused by the epitope spreading of the T-cell reaction from CNS myelin antigen to PNS myelin antigen. Our previous report of peripheral blood T-cell activity in MS showed that the number of clonally expanded T-cell receptor V*β*s increases accompanied by an increase in the expanded disability status scale (EDSS) [19] in patients with relapsing remitting MS [20]. The increased clonally expanded V*β*s might react against not only CNS myelin antigen epitopes but also against PNS myelin antigen epitopes.

Conversely, although MS is a disease mediated by T-cell immunity, there is a possibility that peripheral neuropathy as a complication of MS has a different mechanism from MS and is mediated by humoral immunity. However, there have been limited reports about the relationship between MS and antiganglioside antibodies [21]. Antimyelin oligodendrocyte glycoprotein (MOG) antibodies, anti-MBP antibodies, and antibodies to the ATP-sensitive inward rectifying potassium channel KIR4.1 have been also reported as useful clinical markers of MS [22, 23]. However, these antibodies are not related to peripheral neuropathy as a complication of MS. Genetic contribution to the pathogenic mechanism of MS is widely studied, and results of genome-wide association studies (GWAS) have been reported [24, 25]. One report showed that patients with multifocal motor neuropathy had high frequencies of HLA-DRB1*15 which is known as a risk allele for MS [26], and another report showed that the frequency of HLA-DRB1*15 polymorphism is not associated to chronic dysimmune polyneuropathy [27]. Genetic preponderance for the peripheral involvement to MS is still unclear. 

Interferon beta-1b has been suspected to develop peripheral demyelinating diseases in MS patients [28–30]. Glatiramer acetate (GA) is effective in MS, and intraperitoneal use of GA in experimental autoimmune neuritis significantly ameliorated the severity of disease in a previous report [31]. However, there is another report of development of Guillain-Barre syndrome in a patient with MS during treatment with GA [32]. Thus, the effect of GA for peripheral neuropathy is controversial. In our 6 Japanese MS patients, only one patient received disease-modifying therapy. Thus, we thought that peripheral neuropathy as a complication of MS was not related to disease-modifying drugs. Our study included many untreated MS patients because only interferons beta-1b and beta-1a were approved in Japan for this study period, and currently available disease-modifying therapy is extremely limited compared with western countries. 

One (3.6%) of the 28 NMO patients showed peripheral neuropathy at the same time of NMO relapse. This clinical course is similar to those in two previous reports about the characteristics of peripheral neuropathy as a complication of NMO [7, 8]. Aimoto et al. [7] reported that demyelination of bilateral optic nerves, spinal cord, and peripheral nerves occurred at the same time in Devic's disease. A sural nerve biopsy showed a few demyelination and segmental remyelination. Although they suggested a possibility of common pathogenetic mechanisms in both the central and the peripheral nervous systems, an examination of anti-AQP-4 autoantibody was not performed. Kitada et al. reported [8] that longitudinally extensive transverse myelitis with anti-AQP-4 autoantibody and demyelinating changes in NCS were involved simultaneously in NMO spectrum disorder. Thus, we believe that there is a group of NMO who were affected simultaneously in the central and peripheral nervous tissues. However, the cause is not clear. Because anti-AQP-4 antibody was discovered in the serum of NMO patients as a favorable diagnostic marker, autoantibodies might be associated with peripheral neuropathy in NMO patients. However, antiganglioside antibodies were negative in a previous case report of NMO with peripheral neuropathy [8]. Moreover, anti-AQP-4 antibody cannot cause peripheral neuropathy because AQP-4 is a cell membrane water channel, that is, expressed at the astrocyte foot processes, and there are no astrocytes in the PNS. Undetermined humoral factors other than anti-AQP-4 autoantibody were suggested to cause peripheral neuropathy in NMO [8]. Conversely, it is possible that the pathogenesis of peripheral neuropathy as a complication of MS and NMO is a common mechanism of T-cell immunity. Peripheral blood T-cells were reported to be stimulated against major myelin proteins, that is, MBP, proteolipid protein (PLP), and MOG, in anti-AQP-4 antibody-positive NMO patients, and T-cell lines derived from NMO patients showed inter- and intramolecular epitope spreading [33]. Moreover, peripheral blood T-cell activity of NMO is upregulated compared to MS, especially against AQP-4 and PLP [20, 34]. Possibility exists that peripheral neuropathy as a complication of NMO is caused by the epitope spreading of the T-cell reaction from CNS myelin antigen to PNS myelin antigen as well as MS.

## 5. Conclusions

The present study showed that the frequency of abnormal NCS findings might exhibit no significant differences between MS and NMO, although the cause and pathophysiology of peripheral neuropathy were different in MS and in NMO. 5.2% of MS patients fulfilled the criteria for CIDP. 3.6% of NMO patients showed peripheral neuropathy at the same time of NMO relapse, as in previous reports, although CIDP was not seen in NMO. 5.8% of MS and NMO patients were complicated with neuropathy caused by concomitant conditions such as type 2 DM and Sjögren's syndrome.

## Figures and Tables

**Table 1:** Clinical characteristics associated with CNS demyelinating diseases of 9 patients with peripheral neuropathy.

Patient no.	Sex	MS or NMO	Age at onset	EDSS	Lesions determined by MRI (lesion number)	Number of relapses	OB	AQP-4	DMT
1	F	MS	28	3.5	Cerebrum and brainstem (>10),	3	+	−	−
					spinal cord (5)				

2	F	MS	56	6.5	Spinal cord (4)	Unidentified	+	−	−

3	F	MS	21	2.0	Brainstem (1),	2	+	−	IFN
					spinal cord (5)				

4	M	MS	62	7.5	Spinal cord (4)	4	−	−	−

5	F	MS	38	6.0	Cerebrum and brainstem (>10),	Countless	+	N.T.	−
					spinal cord (>10)				

6	F	MS	33	3.0	Cerebrum (>10),	4	N.T.	N.T.	−
					spinal cord (1)				

7	M	NMO	48	9.0	Brainstem (1),	1	N.T.	+	AZP
					spinal cord (1)				

8	F	NMO	43	6.5	Optic nerve (1),	14	N.T.	+	−
					cerebrum and brainstem (>10),				
					spinal cord (1)				

9	F	NMO	64	6.5	Optic nerve (1),	5	−	+	PSL
					cerebrum and brainstem (10),				
					spinal cord (2)				

OB: Oligoclonal IgG bands

AQP-4: Antiaquaporin-4 autoantibody

N.T.: Not tested

DMT: Disease-modifying therapy

IFN: Interferon beta-1b

AZP: Azathioprine

PSL: Prednisolone

**Table 2:** Clinical characteristics associated with peripheral neuropathy of 9 patients with peripheral neuropathy.

Patient no.	Sex	MS or NMO	CIDP	Abnormal nerve determined by NCS	Characteristics of NCS abnormality	Years from onset of MS/NMO to onset of neuropathy	Complication
1	F	MS	Definite	m, u, p, t, s	S, C	10	—
2	F	MS	Definite	m, u, p, t, s	S, C	Unidentified	—
3	F	MS	Probable	p, t, s	S, C, T	13	—
4	M	MS	—	t, s	S, L	4	DM
5	F	MS	—	t	L	17	DM
6	F	MS	—	u, s	F, L	3	DM
7	M	NMO	—	m, t	L, P	1	—
8	F	NMO	—	t, s	C, L	27	SjS
9	F	NMO	—	m, t, s	C, F, L	10	SjS

m: Median nerve

u: Ulnar nerve

p: Peroneal nerve

t: Tibial nerve

s: Sural nerve

S: Nerve conduction velocity slowing

C: Conduction block

T: Temporal dispersion

F: Reduced F-wave occurrence

L: Low amplitude

P: Prolonged duration

DM: type 2 diabetes mellitus

SjS: Sjögren's syndrome.
